# Supplementary material for: A revision of tetrapod footprints from the late Carboniferous of the West Midlands, UK
Source: PeerJ. 2016 Nov 24;4:e2718. doi: 10.7717/peerj.2718 (PMC5126627; doi:10.7717/peerj.2718)
Supplement: Supplemental Information 1 — Table S1. Accession numbers and taxonomic identifications for the specimens and sandstone slabs from Hamstead, West Midlands, UK. [file peerj-04-2718-s001.docx]

| **Specimen Number**  **(BIRUG BU)** | **Slab Number (BIRUG)** | **Slab Number (RAW)** | **Hardaker ID** | **Current ID** |
| --- | --- | --- | --- | --- |
| BIRUG BU5267 | BIRUG 22126 | RAW 1 | *Ichnium sphaerodactylum*, H1 | *Limnopus* isp. |
| BIRUG BU5268 | BIRUG 22125 | RAW 2 | *Ichnium sphaerodactylum*, H1 | *Limnopus* isp. |
| BIRUG BU5269 | BIRUG 22137 | RAW 3 |  | *Dimetropus leisnerianus* |
| BIRUG BU5270 | BIRUG 22136 | RAW 5 | *Ichnium sphaerodactylum*, H1 | *Limnopus* isp. |
| BIRUG BU5271 | BIRUG 22142 | RAW 6 | *Ichnium sphaerodactylum*, H1 | ? *Limnopus* isp. |
| BIRUG BU5272 | BIRUG 22138 | RAW 7 | *Ichnium sphaerodactylum*, H1 | *Limnopus* isp. |
| BIRUG BU5273 | BIRUG 22138 | RAW 7 | *Ichnium sphaerodactylum* (minimum), H1b | Tetrapoda indet. (cf. *Limnopus*, cf. *Batrachichnus*) |
| BIRUG BU5274 | BIRUG 22139 | RAW 8 |  | Tetrapoda indet. |
| BIRUG BU5275 | N/A | RAW 9 |  | *Dimetropus leisnerianus* |
| BIRUG BU5276 | N/A | RAW 9 |  | Tetrapoda indet. |
| BIRUG BU3294 | N/A | RAW 9 |  | *Batrachichnus salamandroides* |
| BIRUG BU5277 | BIRUG 22127 | RAW 10 |  | *Dimetropus leisnerianus* |
| BIRUG BU5278 | BIRUG 22127 | RAW 10 | *Ichnium sphaerodactylum*, H1 | *Limnopus* isp. |
| BIRUG BU5279 | BIRUG 22129 | RAW 11 |  | *Dimetropus leisnerianus* |
| BIRUG BU5280 | BIRUG 22130 | RAW 12 |  | *Batrachichnus salamandroides* |
| BIRUG BU5281 | BIRUG 22140 | RAW 13 | *Ichnium gampsodactylum*, H5 | *Dromopus lacertoides* |
| BIRUG BU5282 | BIRUG 22131 | RAW 14 |  | *Dromopus lacertoides* |
| BIRUG BU5283 | BIRUG 22131 | RAW 14 | *Ichnium sphaerodactylum*, H1a | Tetrapoda indet. |
| BIRUG BU5284 | BIRUG 22133 | RAW 15 |  | *Limnopus* isp. |
| BIRUG BU5285 | N/A | RAW 16 |  | *Batrachichnus salamandroides* |
| BIRUG BU5286 | BIRUG 22134 | N/A |  | *Limnopus* isp. |
| BIRUG BU5287 | N/A | RAW 17 |  | Arthropoda indet. |
| BIRUG BU5288 | N/A | RAW 18 |  | Arthropoda indet. |
| BMAG 19/14 |  |  |  | *Limnopus* isp. |
